# Elimination of fosfomycin during dialysis with the Genius system in septic patients

**DOI:** 10.1038/s41598-021-91423-9

**Published:** 2021-06-08

**Authors:** T. Dimski, T. Brandenburger, M. Janczyk, T. Slowinski, C. MacKenzie, D. Kindgen-Milles

**Affiliations:** 1 Department of Anesthesiology, University Hospital Düsseldorf, Heinrich Heine University Düsseldorf, Moorenstr. 5, 40225 Düsseldorf, Germany; 2 Eurofins Laborbetriebsgesellschaft Gelsenkirchen GmbH, Gelsenkirchen, Germany; 3 Department of Nephrology, University Hospital Charité, Berlin, Germany; 4 Institute of Medical Microbiology and Hospital Hygiene, University Hospital Düsseldorf, Heinrich Heine University Düsseldorf, 40225 Düsseldorf, Germany

**Keywords:** Infectious diseases, Bacterial infection

## Abstract

To assess fosfomycin (FOS) elimination in patients with sepsis and acute kidney injury (AKI) undergoing slow-extended daily dialysis (SLEDD) with the Genius system in a prospective observational study. After ethics committee approval ten patients with sepsis and AKI stage 3 underwent daily SLEDD sessions of eight hours. FOS was applied i.v. at doses of 3 × 5 g per day. FOS serum levels were measured pre- and post hemofilter before, during, and after SLEDD sessions, and instantaneous clearance was calculated. In five of the patients, we analyzed FOS levels after the first dose, in the other five patients serum levels were measured during ongoing therapy. FOS was eliminated rapidly via the hemofilter. FOS clearance decreased from 152 ± 10 mL/min (start of SLEED session) to 43 ± 38 mL/min (end of SLEDD session). In 3/5 first-dose patients after 4–6 h of SLEDD the FOS serum level fell below the EUCAST breakpoint of 32 mg/L for Enterobacterales and Staphylococcus species. In all patients with ongoing fosfomycin therapy serum levels were high and above the breakpoint at all times. FOS toxicity or adverse effects were not observed. FOS serum concentrations exhibit wide variability in critically ill patients with sepsis and AKI. FOS is eliminated rapidly during SLEDD. A loading dose of 5 g is not sufficient to achieve serum levels above the EUCAST breakpoint for common bacteria in all patients, considering that T > MIC > 70% of the dosing interval indicates sufficient plasma levels. We thus recommend a loading dose of 8 g followed by a maintenance dose of 5 g after a SLEDD session in anuric patients. We strongly recommend therapeutic drug monitoring of FOS levels in critically ill patients with AKI and dialysis therapy.

## Introduction

Sepsis is a common complication and a major cause of mortality in critically ill patients^1^. The most important treatment is focus control and rapid application of antibiotics^2^. Antibiotic drug dosing must be appropriate to achieve a serum concentration above the minimal inhibitory concentration (MIC) for common bacteria. Unfortunately, an increasing number of infections are caused by multi-drug resistant (MDR) bacteria rendering the choice of antibiotics difficult. The decreasing number of effective antibiotics and a paucity of new antimicrobials has led to the revival of “old” antibiotics with preserved activity against MDR bacteria. One of these is Fosfomycin (FOS). FOS is a bactericidal antibiotic that exerts its activity by blocking bacterial cell wall synthesis. It has a broad activity against gram positive bacteria (including methicilline resistant staphylococcus aureus (MRSA)) and gram negative bacteria such as Pseudomonas and Enterobacterales including selected strains of extended-spectrum-β-lactamase (ESBL)-producing bacteria^3^. FOS is a hydrophilic drug with a low weight of 138 g/mol. It´s protein binding is negligible^4^ and it is well distributed in body fluids and various tissues^5,6^. FOS is eliminated via glomerular filtration and total clearance is correlated to creatinine clearance^4^, while the extrarenal elimination is negligible. For critically ill patients, namely those being treated with renal replacement therapy (RRT) almost no data for FOS are available. This is surprising because AKI occurs in 22–53% of septic patients, and approx. 30–50% of these finally need RRT^7^. These patients are at risk of overdosing which may cause drug-related adverse effects while underdosing may increase mortality and bacterial resistance.

In many ICUs to date, patients with severe AKI are treated with slow-extended daily dialysis therapy (SLEDD). This technique delivers effective dialysis doses and provides adequate hemodynamic stability in patients with mild to moderate vasopressor doses^8^. The use of SLEDD thus is safe, easy to handle, and cost effective when compared to continuous renal replacement therapies^9^ but data on FOS elimination via SLEDD are not available.

We here investigate the effect of an eight hours SLEDD therapy on FOS serum levels in critically ill patients with sepsis and AKI stage III. From these data, recommendations on adequate drug dosing can be made.

## Methods

This prospective observational study was approved by the ethics committee of Heinrich-Heine University (#3413) and registered in the university study register. Patients were included after written informed consent. Inclusion criteria were sepsis or septic shock according to the Sepsis-3 definition^10^, AKI Stage-III according to the KDIGO classification^11^, intravenous application of FOS, and RRT with SLEDD. Exclusion criteria were refusal of participation, pregnancy, age under 18 years and intolerance or allergy against FOS.

For clearance calculation serum FOS concentration was measured before and after the dialyzer. In 5 patients we measured serum levels after the first infusion of FOS. Another 5 patients were studied during ongoing FOS therapy, i.e. they had received at least two doses before they were included. A dose of 5 g of FOS (Infectofos 5 g) was infused over a period of 30 min every 8 h. For the patients with ongoing FOS therapy baseline levels were measured before administering the pre-SLEDD dose. FOS serum levels were measured at start of SLEDD and after 15, 30, 60, 120, 240 and 480 min. FOS levels were measured at the Hygieneinstitut Gelsenkirchen, Germany using a validated LC–MS/MS technique (Chromolith Performance RP-18e100-3 column, Merck). The lower limit of quantification was 0.5 mg/L.

SLEDD was performed using the Genius system (Genius, Fresenius Medical Care, Bad Homburg)^12^ and a polyamix hemofilter (Polyflux140H, Baxter Deutschland, Unterschleissheim, surface area 1.4 m^2^, ultrafiltration coefficient 60 mL/h/mmHg, sieving coefficient beta2-microglobulin 0.7). The Genius system is a single-pass batch dialysis system which uses ultrapure sterile dialysis fluid. Per session, 90 L of individually prepared dialysis fluid are available. In this study, a SLEDD session was prescribed to last 8 h. Both blood and dialysate flow were 190 mL/min. All patients received unfractionated heparine as a continuous infusion to prevent clotting.

We calculated hemofilter clearance over the dialysis session using the following formula:

$$ {\text{Clearance}}\;\left[ {{\text{ml}}/{\text{min}}} \right] = \left( {{\text{fosfo }}\left[ {{\text{prefilter}}} \right] - {\text{fosfo }}\left[ {{\text{postfilter}}} \right]} \right) \times {\text{blood}}\;{\text{flow}}\;\left[ {{\text{ml}}/{\text{min}}} \right]/{\text{fosfo}}\;\left[ {{\text{prefilter}}} \right] $$

We documented demographic data, source of infection and data from microbiology. Major laboratory parameters as well as hemodynamics and norepinephrine dose were compared before and after SLEDD therapy.

### Ethics approval

The study has been approved by the ethics committee of the University of Duesseldorf (#3413) and registered in the university study register and has therefore been performed in accordance with the ethical standards laid down in the 1964 Declaration of Helsinki and its later amendments.

### Consent to participate

All participants were included into the study after written informed consent.

## Results

We included 10 patients (age 71 ± 15 years, 7 male/3 female, 175 ± 5 cm, 76 ± 7 kg). All patients fulfilled criteria of severe sepsis or septic shock according to the Sepsis 3 Definition^10^, and all were in AKI stage III according to the KDIGO guidelines^11^. The source of infection was abdominal (5), pneumonia (2), soft tissue (2) and unknown focus (1).

All patients received FOS combined with another antibiotic agent (imipenem/cilastatin 3, third generation cephalosporin 3, tigecyclin 2, vancomycin 1, linezolid 1, and aminoglycosides 2). In nine patients, multi-resistant bacteria were identified including Pseudomonas aeruginosa, Klebsiella oxytoca, Enterobacterales sp. and MRSA (Table 1). One patient suffered from sepsis of unknown focus without detection of bacteria. (Table 1).

**Table 1:** Demographics, source of Infection and antibiotic medication.

Patient	1	2	3	4	5	6	7	8	9	10
Age	26	67	77	83	76	80	80	71	75	75
Height (cm)	172	172	175	180	163	178	178	180	180	175
Body weight (kg)	60	72	70	80	90	75	75	78	78	80
Bacteria	none	S. epid	K. oxytoca, P. aeruginosa	E. cloacae	MRSA	P. aeruginose, E. faecium	P. aeruginosa	E. coli	E. coli	none
Antibiotics	Gentamycin/Ceftazidime/Fosfomycin	Vancomycin/Ceftazidime/Fosfomycin	Imipenem/Fosfomycin	Imipenem/Fosfomycin	Linezolid/Fosfomycin	Tigecyclin/Fosfomycin	Tigecyclin/Fosfomycin	Imipenem/Fosfomycin	Imipenem/Fosfomycin	Tigecyclin/Fosfomycin
Inf. source	Focus unknown	Focus lung	Focus soft tissue	Focus abdominal	Focus lung	Abscess intraabdominal	Abscess	Peritonitis	Peritonitis	hip infection
Hospital survival	Yes	Yes	Yes	No	No	No	No	Yes	Yes	No

**Figure 1:**
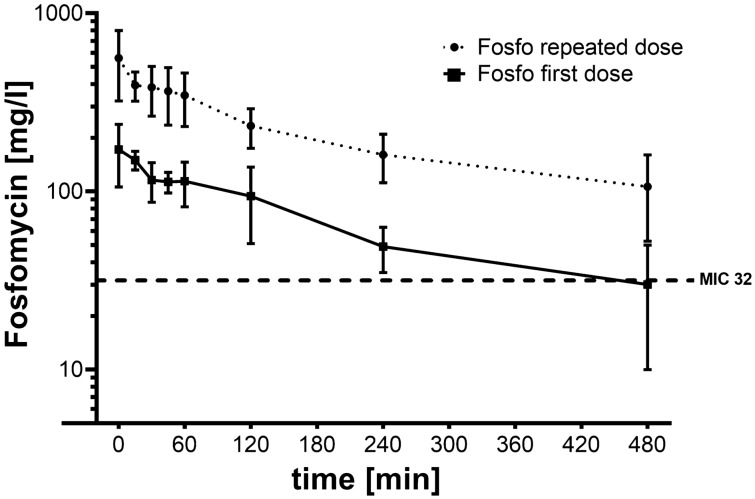
Fosfomycin serum levels in 10 patients during slow-extended dialysis. Squares show serum levels in patients after the first application of fosfomycin (5 g). Circles show serum levels in patients with ongoing therapy, after at least two doses of fosfomycin (5 g) (data from 10 patients, mean ± SD). (Figure created with GraphPad PRISM, version 7.05, www.graphpad.com).

The serum levels of FOS are shown in Fig. 1. In patients that received a first dose of 5 g FOS, mean serum levels peaked at 172 ± 66 mg/L and decreased rapidly during SLEDD therapy. After 240 min the mean serum levels were close to the EUCAST breakpoint of 32 mg/L in all patients, and below the EUCAST breakpoint in 2 patients. At the end of the SLEDD session, the mean serum level was 30 ± 20 mg/L and thus below the EUCAST breakpoint.

In patients with at least two preceeding doses of FOS serum levels were already 320 ± 83 mg/L before the application of the pre-iHD FOS dose. After application of the next dose before iHD serum levels peaked at 561 ± 239 mg/L, decreased rapidly during the SLEDD session, but remained well above the EUCAST breakpoint in all patients until the end of SLEDD therapy.

**Figure 2:**
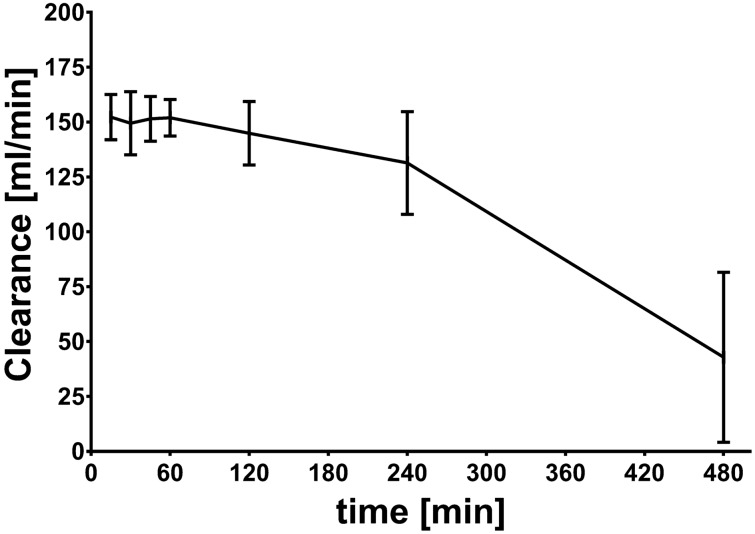
Fosfomycin clearance. Fosfomycin clearance via the hemofilter during slow-extended dialysis (data from 10 patients, (mean ± SD) (Figure created with GraphPad PRISM, version 7.05, www.graphpad.com).

The FOS clearance at the start of SLEDD session was 152 ± 10 mL/min. Clearance remained high for approx. 240 min but then started to decrease to 43 ± 38 mL/min towards the end of the dialysis session (Fig. 2).

The data for serum parameters and hemodynamics before and after the SLEDD therapy are shown in Table 2. In brief, SLEDD therapy was effective in decreasing urea (urea reduction rate 54%) and creatinine (reduction rate 69%) and maintaining a neutral pH. during SLEDD therapy neither heart rate, nor blood pressure or norepinephrine dose changed significantly.

**Table 2:** Serum chemistry and hemodynamic parameters before and after slow-extended daily dialysis (data from 10 patients, mean ± SD).

Parameter	pre SLEDD	post SLEDD
Creatinine [mg/dL]	1.3 ± 0.4	0.9 ± 0.3
Urea [mg/dl]	93.6 ± 36.1	50.8 ± 16.4
Urine output (mL/24 h)	1874 (± 1335)	
pH	7.4 ± 0.1	7.4 ± 0
Potassium [mmol/L]	4.6 ± 0.4	4.5 ± 0.3
Sodium [mmol/L]	144.2 ± 6.6	138.9 ± 4.1
Magnesium [mmol/L]	0.9 ± 0.1	0.9 ± 0.1
Phosphate. anorg. [mmol/L]	1.2 ± 0.4	0.9 ± 0.3
Temperature [C]	37.2 ± 1	36.7 ± 1
Heart rate [bpm]	91.3 ± 16.3	93.8 ± 13.6
RR systolic [mmHg]	127 ± 12.6	133.3 ± 22.3
RR diastolic [mmHg]	60.4 ± 14.3	61 ± 5.6
Norepinephrine [µg/kg/Min]	0.1 ± 0.1	0.1 ± 0.1
Lactate [mmol/L]	1.3 ± 0.7	1.3 ± 0.6
Thrombocytes (× 1000/µL)	266.3 ± 118.8	252.9 ± 117
PTT (sec.)	42.2 ± 9.3	43.2 ± 14.0
Albumin (g/dL)	2.4 ± 0.4	2.1 ± 0.1

## Discussion

We here show that in patients with acute kidney injury and renal replacement therapy with slow-extended daily dialysis FOS is eliminated rapidly with a high clearance. We recommend a high loading dose and therapeutic drug monitoring to achieve and maintain effective serum levels.

FOS is an “old” antibiotic which has retained significant activity against a variety of multi-drug resistant gram-negative and gram-positive bacteria including MRSA^3^. Therefore, FOS is regularly used in combination with other antibiotics to treat severe infections caused by MDR bacteria in ICU patients. Critically ill patients with sepsis or septic shock often require RRT for AKI. Unfortunately, data on elimination of FOS via different RRT techniques are limited. FOS is a hydrophilic low molecular weight antibiotic with almost negligible protein binding rate. Under physiological conditions FOS is eliminated unchanged exclusively via glomerular filtration^13^. Therefore, a decrease of glomerular filtration rate directly decreases FOS clearance and in anuric patients the elimination of FOS occurs via RRT, only.

For ICU patients with moderate hemodynamic impairment, SLEDD therapy has been introduced in clinical practice^12^. SLEDD therapies target at a treatment duration of 8–12 h per day. Clinical studies show effective solute and volume control and good hemodynamic stability even with mild or moderate vasopressor demand^14^.

Unfortunately, almost no data on removal of FOS during SLEDD are available. Kielstein et al. described FOS removal in two patients undergoing iHD and SLEDD. In a severly underweight patient of 40 kg body weight a dose of 3 g of FOS yielded a peak concentration of 106 mg/L which decreased to 49 mg/L following 6 h of SLEDD therapy with the Genius system. In another underweight patient of 49 kg body weight a dose of 3 g four hours prior to dialysis yielded high serum levels which were decreased rapidly during haemodialyis, requiring an additional dose after dialysis. No other data have been published in the context of SLEDD therapy and these two cases do not allow any meaningful dose recommendation^15^.

We here show that following infusion of 5 g FOS in critically ill patients with normal body weight the drug is removed rapidly during a standard SLEDD session. After eight hours SLEDD for both the first as well as the repeated application of FOS the serum levels were reduced by approx. 81%.

Of note, there is an ongoing discussion whether FOS displays a concentration (AUC/PD) or time (T > MIC/PD) dependent bactericidal activity. Bactericidal activity against strains of E.coli, Proteus mirabilis and Streptoccus pneumonia is concentration dependent whilst there is time dependent killing activity against Staphylococci and Pseudomonas species. The EUCAST defines the susceptibility breakpoint as ≤ 32 mg/L for *Enterobacterales* and *Staphylococcus* spp. for intravenous formulation^16^.

In patients in whom FOS therapy was started with a first dose of 5 g, serum levels decreased to an extent that after 4 h in 2 out of 5 patients and in almost every patient after 8 h serum levels were below EUCAST breakpoint level of 32 mg/L. However, T > MIC was more than 70% for all patients in this group. In patients that had received at least two doses of FOS before SLEDD therapy, serum peak levels were high and well above the EUCAST breakpoint also after 8 h of SLEDD.

Initial FOS clearance via the hemofilter was high (152 ± 10 mL/min) and remained stable for the first four hours of therapy. We observed a decrease of FOS clearance during the SLEDD sessions which was most pronounced towards the end of the dialysis session, i.e. after 8 h. This appears surprising at first glance because FOS is a small solute with a molecular weight of approx. 138 Da which is close to the molecular weight of creatinine (113 Da) or urea (60 Da). Unfortunately, we did not calculate instantaneous urea or creatinine clearance during the dialysis so that we cannot compare clearance rates of these solutes at the end of the session. However, we observed a urea reduction rate of 54% and a creatinine reduction rate of 69%. The decrease of FOS levels at the end of the dialysis session was close to 80% in those with a first dose. Thus, regarding the decrease of serum levels elimination of FOS was at least as effective or even more effective as elimination of urea and creatinine. The decrease of FOS clearance at the end of the dialysis session can be explained by a specific technical feature of the Genius System. Towards the end of the dialysis—with almost all fresh dialysate being spent—a small amount of fresh dialysate can mix with already spent dialysate. If instantaneous clearance is calculated from samples taken at this time point—which we did targeting to get a clearance calculation at the end of treatment—than the calculated clearance may be inaccurate, i.e. to low^17^. We did not consider this technical aspect while planning this study. However, the most important observation is the reduction of FOS serum levels which is not affected by this effect. Nevertheless, in critically ill patients, a decrease in filter patency is often observed for intermittent as well as continuous treatment modes and also for so-called high cut-off membranes^18^.

In the light of these observations, it can be challenging to prescribe the optimal FOS dose in patients with sepsis and SLEDD therapy. In addition, we cannot conclude from our data whether the fall below EUCAST breakpoint levels in some patients is relevant for outcome. However, for patients starting iv FOS treatment before a SLEDD session, a starting dose of 5 g FOS may result in low plasma levels for several hours. Therefore, a loading dose of 8 g might be better suited considered to avoid ineffective serum levels towards the end of the dialysis session.

Maintenance dosing of FOS for the next days is dependent on any residual renal function and the frequency and duration of SLEED sessions. In anuric patients with no residual renal function and long interdialytic time periods repeated doses of FOS may lead to a rapid accumulation. In contrast, in patients with daily SLEDD therapy and/or residual renal function the risk of underdosing is obvious. Almost all patients in this study had been on CRRT previous to SLEDD. An endogenous creatinine clearance was not measured because no steady state condition was present.

Therefore, and in the light of the discussion regarding FOS pharmacodynamics it would be a practical approach to use a high loading dose of 8 g to achieve and maintain sufficiently high serum levels following the first application in all patients. For maintenance therapy, a lower dose of 5 g following a full SLEDD session seems adequate.

Of note, our conclusions are limited due to the number of SLEDD therapies performed. However, this is the first series investigating FOS elimination during SLEDD. Our data show that there is an elimination of FOS with the Genius SLEDD which is relevant for drug dosing. Furthermore, there is a high interindividual variability of achieved serum levels of FOS. Even a much larger number of treatments thus would not allow a general recommendation valid for every patient. This is also true for any specific calculation of membrane characteristics, i.e. one could suggest to calculate KoA coefficients. However, due to the specific technical characteristics of the GENIUS System such data cannot be used in any other treatment mode. Therefore, we did not perform such calculations but we recommend individual therapeutic drug monitoring for FOS in patients undergoing dialysis.

Since data on FOS elimination are sparse, our recommendations can only be compared to a study by Gattringer et al*.* who studied FOS elimination after one single dose in patients undergoing continuous venovenous haemofiltration. This group recommended a dose of 8 g of FOS every 12 h as appropriate and safe^19^. Unfortunately, this group did not study further FOS levels during ongoing treatment. Nevertheless, at least regarding recommendations for a loading dose their data are in line with our observations. Finally, severe side effects of FOS therapy such as hypokalemia and hypernatremia were not observed in our study.

In conclusion, a practical and safe approach for FOS dosing would be to use an initial loading dose if 8 g followed by a maintenance dose of 5 g after a complete SLEDD session in anuric patients. However, given the difficulty to predict any individual dose–response relation, we strongly recommend to monitor FOS serum levels in critically ill patients with intravenous FOS therapy and renal replacement therapy.

## Data Availability

Data will be made available on reasonable request.
